# Spontaneously Recycling Synaptic Vesicles Constitute Readily Releasable Vesicles in Intact Neuromuscular Synapses

**DOI:** 10.1523/JNEUROSCI.2005-21.2022

**Published:** 2022-04-27

**Authors:** Yoshihiro Egashira, Ayane Kumade, Akio Ojida, Fumihito Ono

**Affiliations:** ^1^Department of Physiology, Osaka Medical and Pharmaceutical University, Takatsuki, 569-8686, Japan; ^2^Graduate School of Pharmaceutical Science, Kyushu University, Fukuoka, 812-8582, Japan

**Keywords:** neuromuscular junction, spontaneous neurotransmission, synaptic vesicles, zebrafish

## Abstract

Emerging evidence shows that spontaneous synaptic transmission plays crucial roles on neuronal functions through presynaptic molecular mechanisms distinct from that of action potential (AP)-evoked transmission. However, whether the synaptic vesicle (SV) population undergoing the two forms of transmission is segregated remains controversial due in part to the conflicting results observed in cultured neurons. Here we address this issue in intact neuromuscular synapses using transgenic zebrafish larvae expressing two different indicators targeted in the SVs: a pH-sensitive fluorescent protein, pHluorin, and a tag protein, HaloTag. By establishing a quantitative measure of recycled SV fractions, we found that ∼85% of SVs were mobilized by high-frequency AP firings. In contrast, spontaneously recycling SVs were mobilized only from <8% of SVs with a time constant of 45 min at 25°C, although prolonged AP inhibition mobilized an additional population with a delayed onset. The mobilization of the early-onset population was less temperature-sensitive and resistant to tetanus toxin, whereas that of the late-onset population was more sensitive to temperature and was inhibited by tetanus toxin, indicating that prolonged AP inhibition activated a distinct molecular machinery for spontaneous SV fusion. Therefore, the early-onset population limited to <8% was likely the only source of spontaneous release that occurred physiologically. We further showed that this limited population was independent from those reluctant to fuse during AP firing and was used in both the hypertonic stimulation and the immediate phase of AP-evoked releases, thereby matching the characteristics of the readily releasable pool.

**SIGNIFICANCE STATEMENT** Synaptic vesicles (SVs) are divided into functionally distinct pools depending on how they respond to action potential (AP) firing. The origin of SVs used for spontaneous fusion remains enigmatic despite intensive studies in cultured preparations. We addressed this question in intact neuromuscular synapses and provided two findings. First, prolonged AP inhibition activated a distinct population of fusion, which needs to be distinguished from genuine spontaneous fusion arising from a highly limited fraction. Second, the limited fraction observed early in the AP inhibition period exhibited the characteristics of readily releasable pool in the subsequent round of stimulation. Our study revealed that the origin of spontaneous SV fusion is restricted to the readily releasable pool among the SV pools involved in AP-evoked fusion.

## Introduction

Neuronal communication is primarily mediated by the action potential (AP)-evoked release of neurotransmitters stored in synaptic vesicles (SVs). In addition to this precisely timed signaling, virtually all synapses exhibit spontaneous transmission that seems to occur randomly. Since its discovery in the neuromuscular junction (NMJ) (Fatt and Katz, 1950, 1952), spontaneous transmission has been frequently considered as biological noise. However, recent studies have revealed its crucial roles in synaptic physiology, both in central synapses (McKinney et al., 1999; Sutton et al., 2006) and NMJs (Frank et al., 2006; Choi et al., 2014). To understand its unique roles, differences between the two forms of release have been investigated, focusing on the SV population and molecular fusion machinery.

Several studies have identified presynaptic molecules that predominantly function in one of the release modes. Evoked release required canonical vesicular soluble N-ethylmaleimide-sensitive factor attachment protein receptor (v-SNARE) proteins, whereas a fraction of spontaneous release was regulated by noncanonical v-SNAREs (Hua et al., 2011b; Ramirez et al., 2012; Lin et al., 2020). The mechanisms underlying the two forms of release also differ, at least in part, in voltage-gated Ca^2+^ channels (Williams et al., 2012; Ermolyuk et al., 2013) and Ca^2+^ sensors (Groffen et al., 2010; Yao et al., 2011).

Despite the accumulating evidence for distinct molecular machineries, it is still controversial whether SV populations undergoing spontaneous and evoked fusion are segregated (Truckenbrodt and Rizzoli, 2014; Schneggenburger and Rosenmund, 2015). SVs within a single presynaptic terminal are generally divided into three distinct pools with respect to their responsiveness to stimulations: a readily releasable pool (RRP), recycling pool, and resting pool (Alabi and Tsien, 2012). In addition to classical optical tools, including styryl dye and antibodies against the luminal side of the SV protein, application of genetically encoded probes, such as a pH-sensitive GFP variant pHluorin or a tag protein in cultured neurons, showed that spontaneously fusing SVs originated from the resting pool and are reluctant to fuse during evoked activity (Sara et al., 2005; Fredj and Burrone, 2009; Chung et al., 2010). However, other groups using similar approaches and preparations reported opposing results (Groemer and Klingauf, 2007; Hua et al., 2010; Wilhelm et al., 2010), which indicated that spontaneous fusion originates from the total recycling pool consisting of the immediately mobilizing RRP and the subsequently mobilizing recycling pool.

One potential explanation for these conflicting results is the heterogeneity among neurons in the culture, with regard to their maturity as well as the neurotransmitter. As suggested by Truckenbrodt and Rizzoli (2014), constitutive vesicle fusion required for synaptogenesis occurs actively in immature presynaptic terminals and may complicate the detection of genuine spontaneous SV fusion. Moreover, recent studies have shown that the mechanisms regulating spontaneous fusion are different between glutamatergic and GABAergic synapses (Courtney et al., 2018; Lin et al., 2020), further complicating the interpretation of data. Another model system frequently used in this field is the NMJ, an *in vivo* synapse with a single neurotransmitter (Rizzoli and Betz, 2005). However, a detailed investigation of the origin of spontaneous fusion using genetically encoded probes has not been performed in the NMJs.

Here, we used intact NMJs of larval zebrafish, the hallmark of which is a small-sized bouton with a single release site (Wen et al., 2016b), amenable to the optical assays of SV recycling. Moreover, the patch-clamp technique applicable to small muscle cells enables the resolution of all quantal events with large current amplitudes (Brehm and Wen, 2019), offering an ideal platform to study spontaneous release. In this study, we generated transgenic (Tg) fish expressing two independent indicators of SV recycling, pHluorin and HaloTag. By establishing a quantitative measure of the SV fraction based on HaloTag labeling, we found that AP inhibition activated a distinct population of fusion after 1 h, which needs to be distinguished from the genuine spontaneous fusion arising from a highly limited fraction. We further investigated which of the three major SV pools it originated from.

## Materials and Methods

### Larval zebrafish

Zebrafish were raised and maintained under a 14 h light–10 h dark cycle. Embryos and larvae were maintained at 28°C–30°C in egg water containing 0.006% sea salt and 0.01% methylene blue. Larvae, whose sex was not determined, were used for experiments at 4 or 5 d postfertilization (dpf). All animal procedures were performed in accordance with the institutional guidelines for the care and use of animals.

### Molecular biology

The zebrafish hb9 promoter was used to drive the expression of the advanced tetracycline transactivator (tTAad), specifically in motoneurons. The tTA response element (TRE) composite promoter that drives the bicistronic expression of TagRFP and VpHalo, a fusion protein of zebrafish vesicular GABA transporter (VGAT) with pHluorin and HaloTag, was also cloned into the same plasmid to enable inducible gene expression in a single Tg line. We chose VGAT as a target for fusion based on three reasons: First, unlike SNAREs and Ca^2+^ sensors, a biased role toward either form of fusion has not been reported. Second, the fraction residing on the presynaptic surface membrane is expected to be minimal (Santos et al., 2013). Third, a fusion protein can be designed simply by targeting its C-terminus that faces the SV lumen (Martens et al., 2008). Tg (*hb9:tTAad, TRE:TagRFP-P2A-VpHalo*) fish were generated by coinjection of the purified plasmid (25 ng/μl) and *tol2* transposase mRNA (25 ng/μl) into one-cell stage embryos. The injected embryos were treated with 10 μg/ml doxycycline dissolved in egg water from 6 to 24 hpf to prevent transient expression of an excess amount of exogenous proteins, which may interfere with normal development. DNA encoding zebrafish VGAT (SLC32A1) was amplified via PCR from larval zebrafish cDNA using the following oligonucleotide: a forward primer (5′-cccactagtgccaccatggctacgttaataagaag-3′) and a reverse primer (5′-accgctagcatccggcaggttgtatttg-3′). The PCR products were ligated into the SpeI site of the plasmid vector containing Tol2 arms (Kawakami et al., 2004). The sequence for the hb9 promoter (a generous gift from M. Nonet in Washington University), Tet-Off system (Clontech), TagRFP (Evrogen), pHluorin (Egashira et al., 2015), and HaloTag (Promega) were also cloned into the vector. The Tg (*cmlc2:mcherry, TRE:TeNTlc*) fish that express the tetanus toxin (TeNT) light chain (TeNTlc) in a tTAad-inducible manner were generated using the Tol2 transposon system. DNA encoding TeNTlc was cloned from pGEMTEZ-TeTxLC (Addgene plasmid #32640). The sequences for the myocardium-specific promoter (cmlc2) driving the expression of mcherry were incorporated into the plasmid to allow the screening of Tg fish.

### Synthesis of HaloTag ligand-conjugated dye

The HaloTag amine (O_2_)–Cy5 conjugate ligand (HaloTag ligand–Cy5) was synthesized as follows. EDCI·HCl (6.1 mg, 32 mmol), HOBt·H_2_O (4.9 mg, 32 mmol), and DIEA (18.8 ml, 108 mmol) were added to a solution of Cy5 dicarboxylate (20 mg, 27 μmol) and HaloTag amine (O_2_) ligand (Promega) (12.1 mg, 54 μmol) in dry DMSO (2 ml). The mixture was stirred for 10 h at room temperature. After dilution with 1% aqueous TFA solution, the mixture was purified by reverse-phase HPLC (YMC-Triart C18; 250 × 10 mm ID mobile phase gradient, CH_3_CN (0.1% TFA)/H_2_O (0.1% TFA) = 5/95 → 50/50; linear gradient over 40 min). The fractions were collected and lyophilized to release the HaloTag amine (O_2_)–Cy5 conjugate ligand (5.3 mg, 23%) as a blue powder. MALDI-TOF MS *m/z* calculated for C_41_H_55_ClN_3_O_11_S_2_ [M]^+^ was 864.30, whereas the observed value was 864.35.

HaloTag amine (O_2_)–CypHer5E conjugate ligand (HaloTag ligand–cypHer5E) was synthesized as follows. Triethylamine (1.3 μl) was added to a 20 mm solution of HaloTag amine (O_2_) ligand (Promega) (0.24 mg, 0.91 μmol) in MeOH (1 ml) to adjust the pH at 8.5-9.0. This solution was transferred to a plastic tube containing CypHer5E NHS ester (Cytiva) (1 mg, 1.18 mmol), and the mixture was incubated for 4 h at room temperature. After removal of the solvent by centrifugal evaporation, the residue was dissolved in DMSO (45 μl) to prepare a stock solution of a HaloTag amine (O_2_)–CypHer5E conjugate ligand (∼10 mm). This solution was kept at −30°C and used for the protein labeling experiment after thawing. MALDI-TOF-MS (matrix: CHCA) *m/z* calculated for C_44_H_62_ClN_3_O_12_S_3_ [M + H]^+^ was 956.33, whereas the observed value was 956.63.

### Electrophysiological recordings

Whole-cell voltage-clamp recording at a holding potential of −80 mV was performed from fast muscles at room temperature ranging from 23°C to 27°C, unless otherwise stated. Zebrafish larvae were anesthetized with 0.02% tricaine in standard extracellular solution containing 112 mm NaCl, 2.0 mm KCl, 10 mm HEPES, 10 mm glucose, 2 mm CaCl_2_, and 1 mm MgCl_2_, pH 7.3-7.4. The skin, head, and internal organs were then removed. The fish preparation was transferred to a recording chamber placed under an upright microscope (Olympus) and mechanically fixed by a nylon thread glued to a platinum frame. Fish were continuously perfused with a standard extracellular solution. To record miniature endplate currents (mEPCs), 1 μm TTX was added to the extracellular solution. To record a response to hypertonic stimulation, 500 mm sucrose added to the standard extracellular solution was perfused in the presence of 0.1 μm d-tubocurarine. The patch pipette internal solution contained 120 mm K-methanesulfonate, 5 mm KCl, 5 mm EGTA, and 10 mm HEPES, pH 7.2-7.3. An inline solution heater (Warner Instruments) was used to control the temperature of the extracellular solution. Data acquired with an EPC10 amplifier (HEKA) were digitized at 50 kHz and low-pass filtered at 3 kHz. The series resistance was typically ∼10 mΩ and was not compensated. Recordings with series resistance >15 mΩ or >25% change in series resistance were excluded from the analysis. The mEPC events were detected using a template matching algorithm (Clements and Bekkers, 1997) bundled in NeuroMatic tools (Rothman and Silver, 2018), which was run on Igor Pro software (WaveMetrics). This method enabled the exclusion of mEPCs originating from neighboring muscles because their kinetics were much slower because of their propagation through gap junctions.

### Fluorescent labeling and image analysis

For HaloTag-based SV labeling, preparations were made from VpHalo-expressing Tg zebrafish as described above and incubated with 5 μm HaloTag ligand–Cy5 (HL-Cy5), typically in a 1.5 ml plastic tube at 25°C or 30°C (3-6 fish per tube). To label SVs used for evoked release, fish preparations were preincubated with HL-Cy5 for 2 min and subsequently stimulated with 30 mm high K^+^ (HK) extracellular solution, in which 28 mm NaCl of the standard extracellular solution was replaced with equivalent KCl. To label SVs used for spontaneous release, fish preparations were briefly treated with 1 μm TTX and subsequently incubated with HL-Cy5 in the presence of 1 μm TTX. To label the vesicles used for constitutive fusion, preparations were prelabeled with membrane-impermeable HaloTag AcidiFluor Orange ligand (5 μm) for 15 min in HK solution to mask all SVs present at presynaptic terminals. Although AcidiFluor Orange emits orange fluorescence at acidic pH (pK_a_ = 5.0), it quenches at pH 7.4 (Asanuma et al., 2014) and thus functions as a nonfluorescent ligand in our image acquisition conditions. After thorough washing with the standard extracellular solution, the preparations were labeled with HL-Cy5 in the presence of TTX. To label SV recycled during the hypertonic (500 mm sucrose) stimulation, fish preparations were preincubated with the HL-Cy5-containing solution (∼250 μl) for 2 min in a 15 ml plastic tube and subsequently stimulated by adding an equivalent amount of the 1 m sucrose-containing solution. After 25 or 50 s, stimulation was stopped by diluting the solution with an excess volume of the standard extracellular solution. After labeling with HL-Cy5, the preparations were washed 3 times with standard extracellular solution and fixed with 4% PFA overnight at 4°C. After washing 3 times with PBS, pH 7.4, the preparations were mounted on glass-bottom Petri dishes and observed at pH 7.4 using a confocal microscope (SP8, Leica Microsystems) with a 40× oil immersion objective. Fluorescent spectra of TagRFP, pHluorin, and Cy5 were efficiently separated and detected in photon-counting mode. Images (1024 × 1024 pixels) were acquired from 3 or 4 regions of the ventral trunk in each preparation. For every set of experiments, we prepared 3 or 4 samples, where HaloTags were fully labeled with HL-Cy5. To fully label the HaloTags, fish preparations were fixed and incubated with 5 μm HL-Cy5 in PBS containing 0.1% Triton X-100 (PBST) overnight at room temperature.

Acquired images were analyzed using Fiji/ImageJ software. ROIs ranging from 1.5 to 10 μm^2^ were defined by applying an “analyze particles tool” to a binarized image of pHluorin fluorescence at pH 7.4. This procedure roughly delineated the individual presynaptic boutons as an ROI. The fluorescence intensity of pHluorin (F_pH (pH7.4)_) and Cy5 (F_Cy5_) was measured at the same ROIs after background fluorescence was subtracted using the rolling ball algorithm. F_Cy5_/F_pH (pH7.4)_ was calculated at >60 ROIs in every preparation, and the average intensity was used for statistical analyses. To test the difference in the distributions of F_Cy5_/F_pH (pH7.4)_ at individual ROIs, cumulative histograms obtained from each fish were averaged and subjected to statistical analyses.

For AChR labeling with α-Btx, fish preparations were incubated with 1 μg/ml α-Btx-CF633 in standard extracellular solution for 15 min at room temperature. The preparations were rinsed 3 times with standard extracellular solution for 15 min and fixed with 4% PFA overnight at 4°C. After thorough washing with PBS, the preparations were mounted on glass-bottom Petri dishes and observed under a confocal microscope (SP8, Leica Microsystems).

### Live cell imaging and analysis

Tg fish preparation was placed in an imaging chamber continuously perfused with standard extracellular solution supplemented with 3 μm d-tubocurarine and mechanically fixed as described above. When sequential imaging of pHluorin and cypHer5E was performed, preparations were prelabeled with ∼10 μm HaloTag ligand cypHer5E either by 30 mm HK depolarization for 3 min or by incubation in the presence of 1 μm TTX for 45-60 min, followed by washing with standard extracellular solution for at least 15 min. For HK labeling, the temperature during the wash was increased to 30°C to accelerate SV re-acidification. Fluorescence live imaging was performed using an inverted confocal microscope (LSM 510 Meta, Carl Zeiss) with a 40× (1.2 NA) water immersion objective at room temperature. Laser scanned images (300 × 200 pixels; 768 ms/scan) were acquired every 1 s. Therefore, sequential imaging of pHluorin and cypHer5E resulted in a 0.5 Hz time-lapse for each fluorescence. pHluorin and cypHer5E were excited with 488 nm argon and 633 nm helium–neon lasers, respectively. The emission of pHluorin and cypHer5E was collected using the 505 to 530 nm bandpass and 650 nm long-pass filters, respectively. Before time-lapse imaging, an imaging region was selected based on the TagRFP fluorescence, which was excited with a 543 nm helium–neon laser and collected using a 560 nm long-pass emission filter. For electrical stimulation, 1 ms constant voltage pulses (70 mV) were delivered to motoneurons via a theta glass pipette (20- to 40-μm-tip diameter) filled with the extracellular solution and positioned at the center of the spinal cord within the imaging segment. The timing of electrical stimulation and image acquisition was controlled using a digitizer (Molecular Devices). Solutions used during image acquisition were applied directly to the preparation using a perfusion exchange device (ALA Scientific Instruments) under the control of the digitizer. When the extracellular solution was changed to a solution containing 50 mm NH_4_Cl, where the equivalent NaCl of the standard extracellular solution was replaced with NH_4_Cl, the pH of the extracellular solutions was increased to 8.0. The application of 50 mm NH_4_Cl has been used to neutralize the SV interior, which is acidic in the resting condition (Fernandez-Alfonso and Ryan, 2008). However, the resulting pH critically depends on the initial luminal pH and luminal buffering capacity of the SVs (Egashira et al., 2015, 2016). Thus, the SVs that were trapped in the alkaline (pH 7.4) state because of recycling after bafilomycin A1 (Baf A1) treatment may be further alkalized by NH_4_Cl application, which would result in an overestimation of the maximum fluorescence because pHluorin fluorescence still shows pH responsiveness at pH > 7.4. To avoid this effect, the pH of the extracellular solutions was set to 8.0 to saturate the pHluorin fluorescence. Since physiological experiments in zebrafish NMJ are often performed at pH 7.8 (Wen et al., 2016b), a higher pH should not affect the results.

Acquired time-lapse images were analyzed using Fiji/ImageJ software. Circular ROIs (7 μm diameter) were manually positioned at the center of the boutons observed in the TagRFP image taken in the same FOV and were transferred to time-lapse images of pHluorin and cypHer5E. Another 5 ROIs of the same size were positioned in regions where no boutons were visible, and their average fluorescence was subtracted as a background signal. Because the cypHer5E fluorescence, which is maximal at acidic pH, is not photostable, its photobleaching was corrected. The extent of photobleaching was quantified at each bouton using the same image acquisition protocol, without electrical stimulation. The fluorescent decay was fitted with a double exponential, which was used to correct the photobleaching. Data for <10 boutons from a single experiment were averaged and counted as *n* = 1.

### Swimming analysis

Swimming of zebrafish larvae was recorded with a high-speed camera (Kron Technologies) for 100 ms at 1000 frames/s, and a puff stimulus with a glass pipette (20- to 30-μm-tip diameter) filled with egg water was used to elicit escape behaviors. The puff pipette was positioned immediately above the tip of the fish tail, and positive pressure (30 psi, 20 ms) was applied using a pulse pressure device (Parker Hannifin). The onsets of the image acquisition and pulse pressure were synchronized. Acquired images were analyzed using Fiji/ImageJ software. By manually measuring the head turn angle (θ) for each frame, the head turn speed (dθ) and distance traveled between the frame at 20 and 50 ms were calculated.

### Experimental design and statistical analyses

All experiments were designed to achieve sufficient statistical power, and data were analyzed with WaveMetrics Igor Pro and Microsoft Excel. Sample sizes were not predetermined but conformed to similar studies. All data were represented as mean ± SEM. To compare the means of two independent groups, unpaired Student's *t* test was applied when the two groups had similar variance; otherwise, unpaired Welch's *t* test was applied. To compare three or more groups, one-way ANOVA followed by Bonferroni–Holm correction for multiple comparisons was applied. The Kolmogorov–Smirnov test was used to compare the distributions of the two groups. All statistical tests were two-tailed.

## Results

### Two independent indicators of SV recycling were targeted to SVs at the presynaptic terminal of zebrafish motoneurons

**Figure 1.**
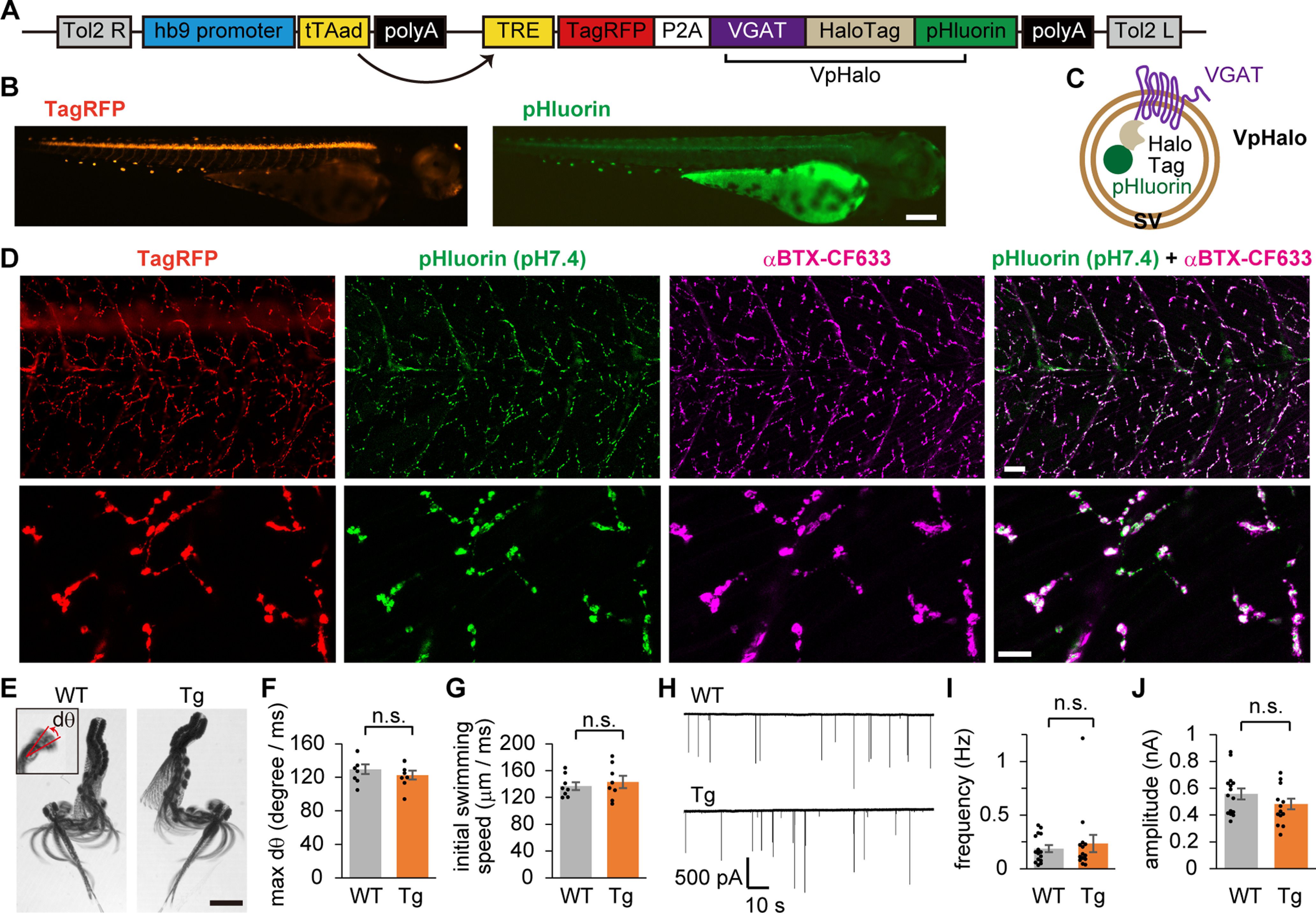
Tg zebrafish expressing two independent indicators of SV recycling in motoneurons. ***A***, Diagram represents the transgene construct introduced in the Tg (*hb9:tTAad, TRE:TagRFP-P2A-VpHalo*) zebrafish. The hb9 promoter drives the expression of the tTAad, which in turn induces the expression of TagRFP and VpHalo through its interaction with the tetracycline response element (TRE) composite promoter. ***B***, Fluorescent stereoscopic images of TagRFP and pHluorin in an anesthetized Tg zebrafish at 4 dpf. Scale bar, 200 μm. ***C***, Diagram represents VpHalo that resides on the SV membrane. ***D***, Confocal microscopic images of TagRFP, pHluorin (pH 7.4), and postsynaptic AChR clusters visualized by α-BTX-CF633 in PFA-fixed Tg zebrafish. Bottom panels, *z*-stack images with higher magnification. Scale bars: 20 μm, 10 μm. ***E***, Swimming was elicited by puff stimulus onto the tail of a 4 dpf zebrafish, and the images were captured at 1000 frames/s. Superimposed images at 4 ms intervals are shown for representative larvae from WT or Tg. Inset, An image with two frames superimposed at 1 ms interval, from which dθ corresponding to the head-turn speed (degree/ms) was measured. Scale bar, 1 mm. ***F***, ***G***, Maximum head-turn speed (***F***) and swimming speed calculated from the distance traveled during initial 30 ms (***G***) in WT (*n* = 8 fish) or Tg (*n* = 8 fish) fish. There was no significant difference between the two groups in head-turn speed (*p* = 0.39, unpaired *t* test) or swimming speed (*p* = 0.58, unpaired *t* test). ***H***, Representative traces of mEPCs recorded from the fast muscle of WT or Tg zebrafish. ***I***, ***J***, Frequency (***I***) and amplitude (***J***) of mEPCs recorded from WT (*n* = 15 cells from 8 fish) or Tg (*n* = 14 cells from 6 fish) fish. No significant difference was found between the two groups in terms of frequency (*p* = 0.60, unpaired *t* test) or amplitude (*p* = 0.20, unpaired *t* test).

To visualize SV recycling accompanied by evoked and spontaneous release, pHluorin and HaloTag were targeted to the luminal side of the SV protein. pHluorin allows live monitoring of activity-dependent SV recycling (Sankaranarayanan et al., 2000), whereas HaloTag visualizes SVs recycled during a given period (Asanuma et al., 2014) by covalently labeling the fused protein (Los et al., 2008). Among SV proteins, we chose the zebrafish VGAT as the optimal target (for the reasons, see Materials and Methods). We generated Tg zebrafish whose motoneurons bicistronically express a reporter red fluorescent protein (TagRFP) and VGAT fused with HaloTag and pHluorin in tandem, which we call “VpHalo.” A transgene containing the Tet-Off inducible system that enhances protein expression driven by a motoneuron-specific promoter (hb9) was integrated into the zebrafish genome with the aid of the Tol2 transposon system (Fig. 1*A*). At 4 dpf, Tg zebrafish exhibited cytosolic TagRFP fluorescence in motoneurons, whereas pHluorin fluorescence was not clearly visible in the living condition (Fig. 1*B*), as expected from its localization in the acidic SV lumen (Fig. 1*C*). When fish were fixed and observed in PBS at pH 7.4 with confocal microscopy, pHluorin fluorescence was visible in bouton-like structures and colocalized with the postsynaptic AChR cluster, indicating that VpHalo was appropriately sorted to SVs in motoneuron terminals (Fig. 1*D*). VGAT is not endogenously expressed in motoneurons and may disrupt its native character. However, locomotion during escape behavior or mEPCs did not change in Tg fish (Fig. 1*E–J*), suggesting that VpHalo expression did not perturb normal motoneuron function.

### VpHalo enabled visualization of AP-evoked SV recycling both by pHluorin live imaging and HaloTag labeling

**Figure 2.**
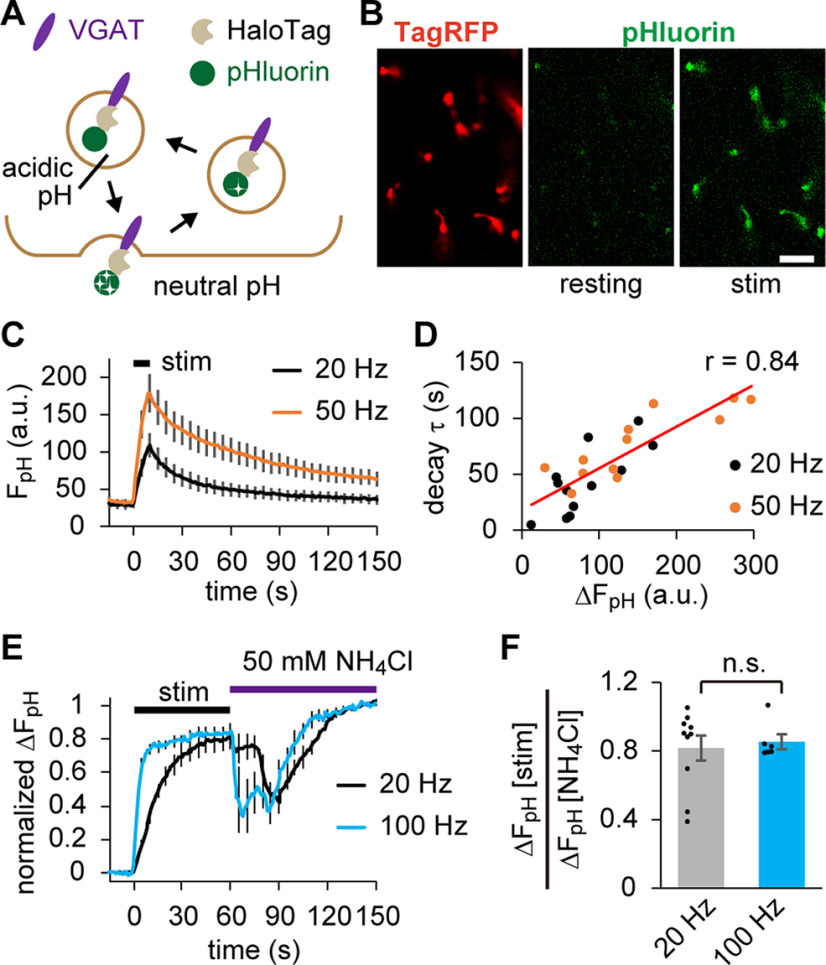
pHluorin live imaging indicated that SVs carrying VpHalo recycle normally in response to APs. ***A***, Diagram represents changes in pHluorin fluorescence during recycling of SVs carrying VpHalo. ***B***, Confocal live images of pHluorin before (resting) and after (stim) APs at 20 Hz for 10 s. The reporter TagRFP fluorescence in the same region is also shown. Scale bar, 10 μm. ***C***, pHluorin fluorescence in response to APs at 20 Hz (*n* = 12 experiments from 7 fish) and 50 Hz (*n* = 12 experiments from 7 fish). ***D***, Relationship between the AP-induced fluorescence increase (ΔF_pH_) and the decay time constant (decay τ). ***E***, pHluorin fluorescence after Baf A1 treatment in response to APs at 20 Hz (*n* = 10 experiments from 10 fish) and 100 Hz (*n* = 6 experiments from 6 fish). To measure the maximal fluorescence, 50 mm NH_4_Cl was subsequently applied. ***F***, ΔF_pH_ [stim] normalized to the fluorescence achieved by NH_4_Cl application (ΔF_pH_ [NH_4_Cl]). No significant difference was observed between the two groups (*p* = 0.72, unpaired *t* test). Error bars indicate ±SEM.

To test whether SVs carrying VpHalo recycle properly during neuronal firing, we performed confocal live imaging of pHluorin fluorescence. Although pHluorin is quenched at resting conditions, it becomes fluorescent when exposed to extracellular neutral pH via SV exocytosis and is again quenched during endocytosis and SV re-acidification (Fig. 2*A*). APs at 20 or 50 Hz frequency lasting for 10 s were elicited by delivering voltage pulses to the spinal cord of a Tg fish, and fluorescence was imaged at boutons located in the ventral part of the stimulated segment. Fluorescence changes in response to APs were observed as expected, and the extent of the change was larger at a higher AP frequency (Fig. 2*B*,*C*). When the increase in fluorescence (ΔF_pH_) was plotted against the time constant of the subsequent decay for each experiment, a high degree of correlation was observed (*r* = 0.84; Fig. 2*D*). These features are consistent with previous studies in mouse NMJs expressing SpH, a pHluorin-fused vesicle-associated membrane protein 2 (VAMP2) (Tabares et al., 2007; Wyatt and Balice-Gordon, 2008), suggesting that VpHalo-carrying SVs are normally recycled. As pHluorin imaging offers a convenient method to separate the total recycling pool and the resting pool under blockade of SV re-acidification (Fernandez-Alfonso and Ryan, 2008), a similar method with a modification in the extracellular pH was used (see Materials and Methods). APs were elicited at 20 or 100 Hz for 30 s after treatment with Baf A1 (4 μm for 2.5 min), a vacuolar-type H^+^ ATPase inhibitor. The observed fluorescence change (ΔF_pH_ [stim]) represents the cumulative amount of exocytosis. After the cessation of APs, 50 mm NH_4_Cl perfusion alkalized all SVs to obtain the maximum signal (ΔF_pH_ [NH_4_Cl]) representing the total amount of SVs (Fig. 2*E*). The total recycling pool was calculated as ΔF_pH_ [stim]/ΔF_pH_ [NH_4_Cl]. With AP frequency >20 Hz, ∼85% of the SVs were mobilized for transmission at the zebrafish NMJs (Fig. 2*F*). The acidification observed at the initial ∼30 s of NH_4_Cl application was somewhat paradoxical. Although the exact mechanism is unclear, this phenomenon was also observed in SpH imaging using a similar preparation (Wen et al., 2016a). Synapses located distant from the perfused solution may undergo a temporary uncompensated phase of acidification induced by slowly penetrating NH_4_Cl.

**Figure 3.**
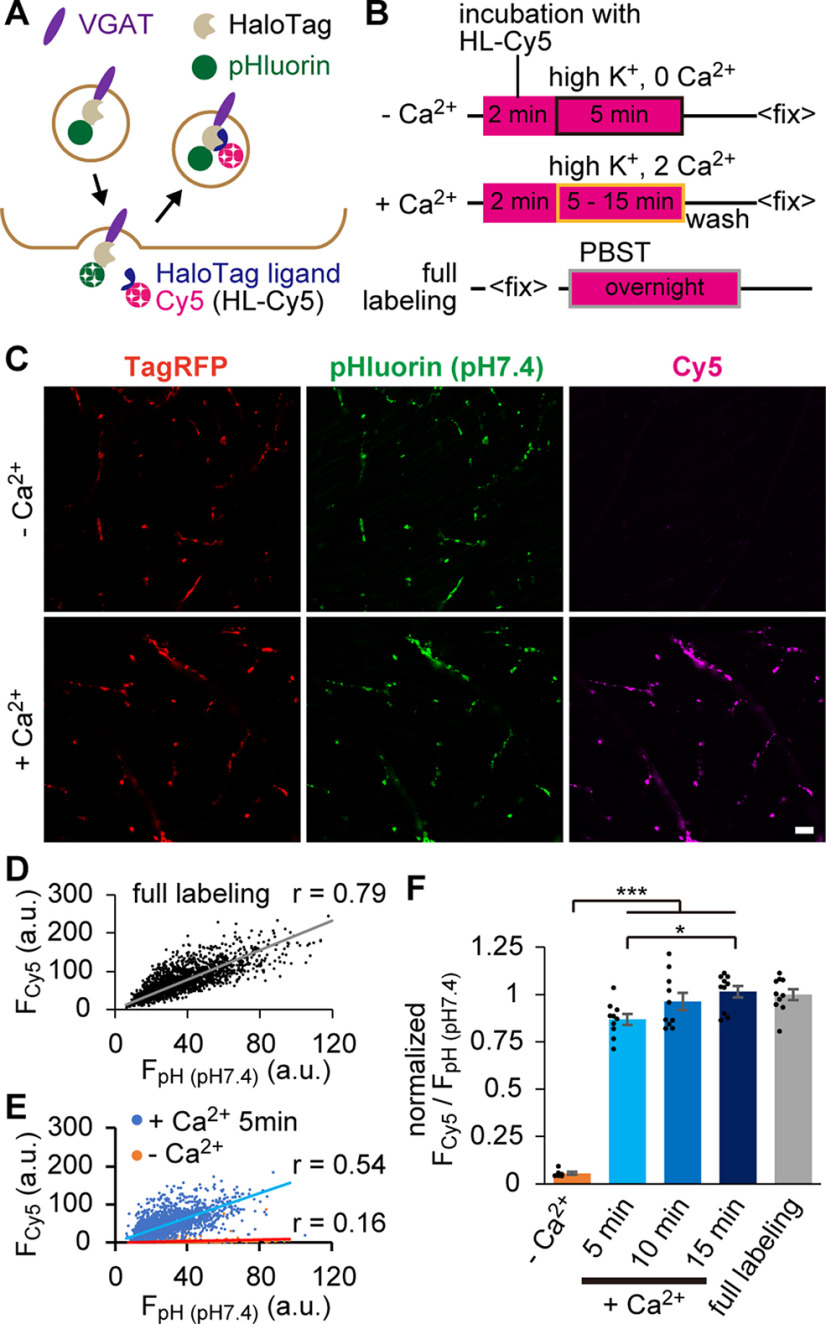
HaloTag-based SV labeling enabled quantifying the fraction of recycled SVs. ***A***, Diagram represents HaloTag labeling with HL-Cy5 during recycling of SVs carrying the VpHalo. ***B***, Diagram represents the timeline of the labeling experiment. Fish preparations were subjected to incubation with HL-Cy5 (magenta square) combined with HK depolarization in the absence (boxed in black; –Ca^2+^) or presence (boxed in orange; +Ca^2+^) of [Ca^2+^]_o_. In parallel, fish after fixation were incubated overnight in PBST containing HL-Cy5 (boxed in gray; full labeling). ***C***, Confocal images of TagRFP, pHluorin (pH 7.4), and Cy5 at the NMJs of fish stimulated by HK for 5 min in the absence (–Ca^2+^) or presence (+Ca^2+^) of [Ca^2+^]_o_. Scale bar, 10 μm. ***D***, ***E***, Relationship between pHluorin fluorescence (F_pH (pH7.4)_) and Cy5 fluorescence (F_cy5_) measured at individual ROI in full labeling (***D***) and HK-stimulated (***E***) conditions. ***F***, Average F_cy5_/F_pH (pH7.4)_ normalized to that of the value in a full labeling condition (*n* = 10 fish). +Ca^2+^ stimulation for 5 min (*n* = 10 fish), 10 min (*n* = 10 fish), and 15 min (*n* = 10 fish) mobilized larger SV fractions than –Ca^2+^ stimulation (*n* = 6 fish). *Adjusted *p* < 0.05, ***adjusted *p* < 0.001, one-way ANOVA followed by Bonferroni–Holm test. Error bars indicate ±SEM.

Next, we performed HaloTag-based labeling of VpHalo, which also enabled the estimation of the pool size of recycling SVs. Incubation of Tg fish with membrane-impermeable HaloTag ligand–Cy5 (HL-Cy5) led to an accumulation of Cy5 dye in recycled SVs because the ligand irreversibly tags VpHalo only when it is exposed to the extracellular space via exocytosis (Fig. 3*A*). Fish were preincubated with HL-Cy5 for 2 min and then stimulated with 30 mm HK solution for 5-15 min (+Ca^2+^) or with Ca^2+^-free HK solution for 5 min (–Ca^2+^), in the presence of HL-Cy5 (Fig. 3*B*). We found strong Cy5 fluorescence (F_Cy5_) only in the +Ca^2+^ condition (Fig. 3*C*), confirming that HaloTag labeling successfully visualized recycled SVs. To estimate the fraction of labeled SVs, we fully labeled VpHalo-carrying SVs in parallel by incubating the fixed fish with HL-Cy5 overnight in PBS containing 0.1% Triton X-100 (PBST) (full labeling; Fig. 3*B*). ROIs were identified based on the pHluorin fluorescence observed at pH 7.4 (F_pH (pH7.4)_), followed by measurement of F_Cy5_ in the ROI. Since F_pH (pH7.4)_ essentially represents the total expression of the Tg probe and showed a high degree of linear correlation with F_Cy5_ in the full labeling condition (*r* = 0.79; Fig. 3*D*), F_Cy5_/F_pH (pH7.4)_ conceivably provides an estimation of the labeled SV fraction. In line with this idea, the correlation between the two fluorescence signals was reduced slightly in the +Ca^2+^ condition (5 min; *r* = 0.54) and considerably in the –Ca^2+^ condition (*r* = 0.16; Fig. 3*E*) compared with the full labeling condition (Fig. 3*D*). The averaged F_Cy5_/F_pH (pH7.4)_ from 3 or 4 fish of full labeling condition was used to normalize the value of individual fish, which represents a proportion of labeled SVs to the total pool (Fig. 3*F*). HK depolarization for 5 min labeled 87 ± 3% of SVs, which roughly corresponded to that mobilized by strong electrical stimulation (Fig. 2*F*). In contrast, HK depolarization over 10 min mobilized virtually all SVs (96 ± 3% for 10 min, 102 ± 3% for 15 min), indicating that SVs carrying VpHalo in zebrafish NMJs fully recycle on strong depolarization.

### Prolonged AP inhibition mobilized a delayed population of spontaneous fusion with distinct temperature sensitivity, suggesting that a limited SV fraction was dedicated to spontaneous fusion in the early phase of AP blockade

**Figure 4.**
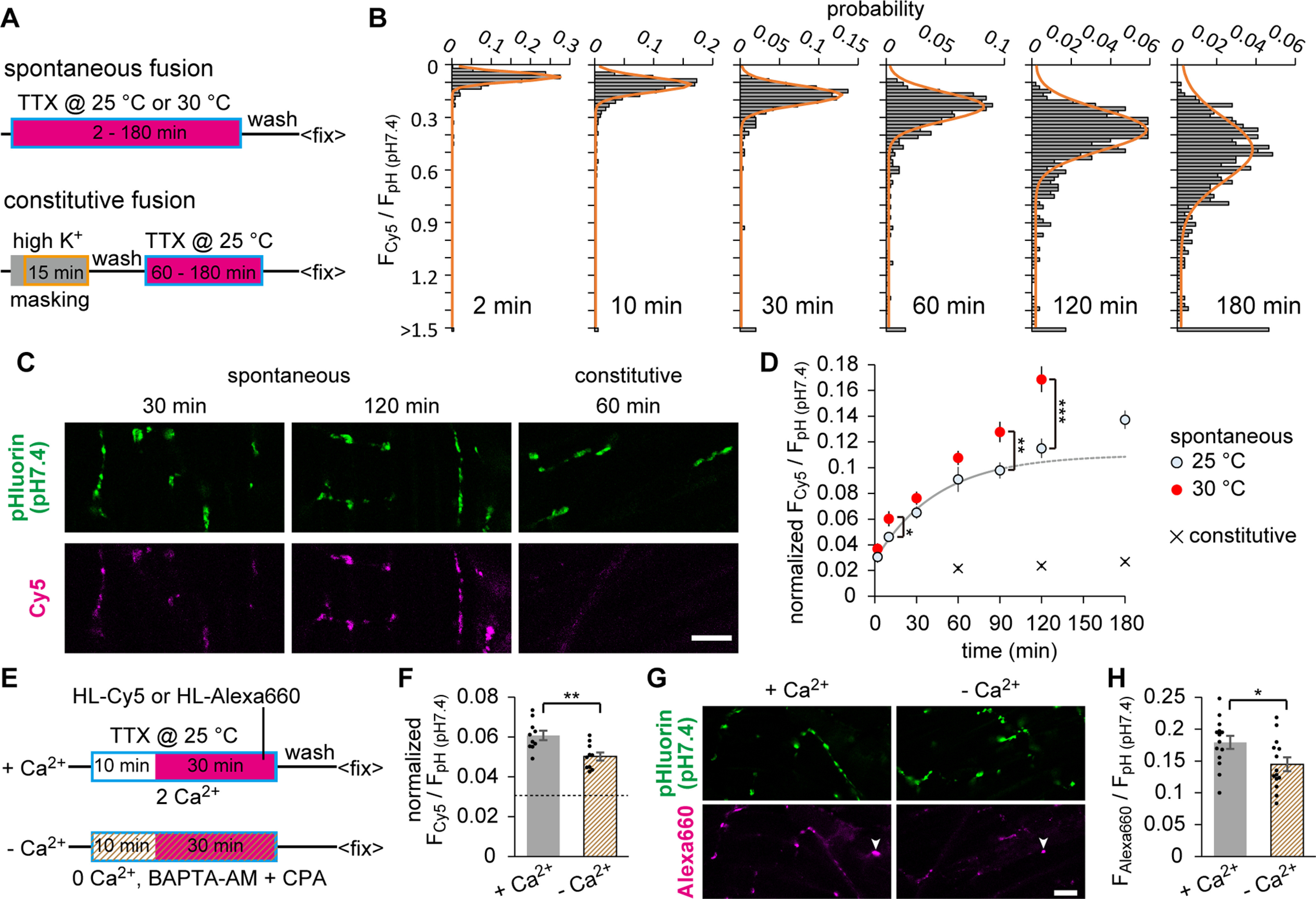
HaloTag labeling of spontaneously recycled SVs suggested two distinct populations of spontaneous fusion. ***A***, Diagram represents the timeline of labeling experiments. Fish preparations were subjected to incubation with HL-Cy5 (magenta square) in the presence of 1 μm TTX for 2-180 min (boxed in blue) at 25°C or 30°C (spontaneous fusion). In measuring the constitutive fusion of transported organelles (constitutive fusion), labeling was performed after HaloTags were first masked by HK depolarization (boxed in orange) in the nonfluorescent ligand (gray square). ***B***, Distribution of F_Cy5_/F_pH (pH7.4)_ measured at individual ROIs. Fish preparations were labeled with HL-Cy5 in the presence of TTX at 25°C for 2 min (483 ROIs), 10 min (538 ROIs), 30 min (547 ROIs), 60 min (556 ROIs), 120 min (526 ROIs), or 180 min (537 ROIs). All histograms were fitted with Gaussian distribution (orange line). ***C***, Confocal images of pHluorin (pH 7.4) and Cy5 at the NMJs. Labeling was achieved by spontaneous fusion for 30 or 120 min, or constitutive fusion for 60 min. Scale bar, 10 μm. ***D***, Time-dependent accumulation of labeled fraction, indicated as F_cy5_/F_pH (pH7.4)_ normalized to the value obtained by full labeling. Spontaneous fusions were labeled at 25°C for 2, 10, 30, 60, 90, 120, and 180 min (*n* = 12, 13, 12, 12, 12, 12, and 8 fish, respectively), or at 30°C for 2, 10, 30, 60, 90, and 120 min (*n* = 9, 12, 12, 12, 11, and 8 fish, respectively). A single exponential fitting to the averaged data up to 90 min at 25°C is shown. Constitutive fusions were labeled at 25°C for 60, 120, and 180 min (*n* = 10 fish). Temperature rise resulted in a significant increase in the spontaneous labeling at 10, 90, and 120 min (*p < 0.05, **p < 0.01, ***p < 0.001, unpaired *t* test). ***E***, Diagram represents the timeline of labeling experiments with or without Ca^2+^. Fish preparations were preincubated with 1 μm TTX (boxed in blue) in 2 mm Ca^2+^ solution (+Ca^2+^) or Ca^2+^ free solution containing 50 μm BAPTA-AM and 50 μm cyclopiazonic acid (–Ca^2+^) for 10 min, and then labeled with HL-Cy5 or HL-Alexa-660 (magenta square) for 30 min at 25°C. ***F***, Spontaneously labeled fraction with HL-Cy5 during 30 min incubation in +Ca^2+^ (*n* = 10 fish) or –Ca^2+^ (*n* = 10 fish) condition. Dashed line indicates the value obtained by 2 min labeling at 25°C shown in ***D***, which reflects background signal, including the surface fraction. The labeled fraction was significantly decreased with the removal of free Ca^2+^ (**p < 0.01, unpaired *t* test). ***G***, Confocal images of pHluorin (pH 7.4) and HL-Alexa-660 at the NMJs. Scale bar, 10 μm. Nonspecific signals (arrowhead), presumably resulting from the dye aggregation, precluded normalization by the pooled data in ***H***. ***H***, Average F_Alexa660_/F_pH (pH7.4)_ in +Ca^2+^ (*n* = 14 fish) or –Ca^2+^ (*n* = 14 fish) condition. Reduction by the Ca^2+^ removal was similar between HL-Cy5 and HL-Alexa-660 (*p < 0.05, unpaired *t* test). Error bars indicate ±SEM.

Shifting our focus next to the spontaneously recycling SVs, we estimated their fraction using pHluorin and Cy5. While pHluorin imaging under the blockade of SV re-acidification has been used to detect spontaneous fusion (Atasoy et al., 2008; Hua et al., 2011b), careful manipulation is required to avoid SV alkalization independent of exocytosis. Thus, an additional independent approach using tagged ligand can potentially provide more reliable readout for detection of low-frequency events (0.01 Hz per synapse; see Discussion). We incubated Tg fish preparations with HL-Cy5 in the presence of TTX for 2-180 min at 25°C or 30°C (Fig. 4*A*). Distributions of F_Cy5_/F_pH (pH7.4)_ measured at individual ROIs in a single experiment performed at 25°C are shown in Figure 4*B*. Two minutes of labeling mostly reflected the surface expression of the probe, including the background signal. All histograms were well fitted with a Gaussian distribution with peaks shifting rightward as the labeling period increased, suggesting that all synapses exhibited spontaneous fusion. The labeled SV fraction was then calculated in each experiment by subjecting 3 or 4 fish to the full labeling, whose averaged F_Cy5_/F_pH (pH7.4)_ was used for normalization. At 25°C, the labeled fraction appeared to saturate within the first 60 min, but started to increase again after 90 min (Fig. 4*C*,*D*). The average data up to 90 min at 25°C were well fitted with a single exponential with a tau of 45 min. This implies that spontaneous fusion first originates from a highly limited population, whereas prolonged AP inhibition activates a second late-onset population. Alternatively, the entire data can be roughly fitted with a single exponential with a slower tau, which predicts a single population with a larger pool size. To test these two possibilities, spontaneous SV labeling was performed at 30°C (Fig. 4*D*). If spontaneous fusion originates from a single component, because of a simple acceleration of the exponential reaction, the temperature rise would result in greater changes in the initial phase than that in the late phase. However, during the initial 60 min, the temperature-dependent acceleration of spontaneous fusion was significant only at 10 min, with a 1.5-fold increase in the initial slopes. In contrast, the temperature increase strongly accelerated spontaneous fusion after 90 min of AP blockade, in which the slope between 90 and 120 min increased 2.4-fold. These results support two distinct populations of spontaneous fusion. The early-onset population was less temperature-sensitive and saturated in 60 min, whereas the late-onset population was more temperature-sensitive and did not saturate within the time window of our experiment. It is important to note that the “late-onset” can be defined only with regard to the AP blockade with TTX. Therefore, the late-onset population presumably appears only after prolonged TTX treatment. It is likely that only the early-onset population underlies the spontaneous SV fusion under physiological conditions. A single exponential fitting to the early-onset population (<90 min) obtained at 25°C allowed us to estimate the fractions of surface expression and the spontaneously recycling SVs. The former was the offset corresponding to 2.8%, and the latter was derived from the curve fitting, corresponding to maximum 8.2% of the full labeling.

The constitutive fusion of trafficking organelles that transport building blocks of presynaptic structures from soma may contribute to the observed F_Cy5_/F_pH (pH7.4)_ (Truckenbrodt and Rizzoli, 2014). By masking VpHalos on all releasable SVs before spontaneous labeling (Fig. 4*A*, see Materials and Methods), we isolated the contribution of constitutive fusion and found it to be negligible under our experimental conditions (Fig. 4*C*,*D*).

Another concern was potential membrane permeability of the HaloTag ligand, which could compromise the sensitivity for detecting spontaneously recycled SVs. We tested this possibility by using HaloTag AlexaFluor-660 ligand (HL-Alexa-660), which is widely accepted as membrane-impermeable, to be compared with HL-Cy5. We acutely removed free Ca^2+^ by applying BAPTA-AM (50 μm) and cyclopiazonic acid (50 μm) in Ca^2+^-free extracellular solution (Fig. 4*E*), which is known to substantially inhibit spontaneous transmission (Courtney et al., 2018). This procedure significantly reduced the SV labeling after 30 min incubation in measurements using either HL-Cy5 (Fig. 4*F*) or HL-Alexa-660 (Fig. 4*H*). Based on the similar extent of inhibition between these two dyes, we reason it is unlikely that HL-Cy5 can permeate the membrane and label nonrecycled SVs.

### mEPC events in contrast to SV labeling relied more on repeated reuse than on virgin exocytosis of SVs

**Figure 5.**
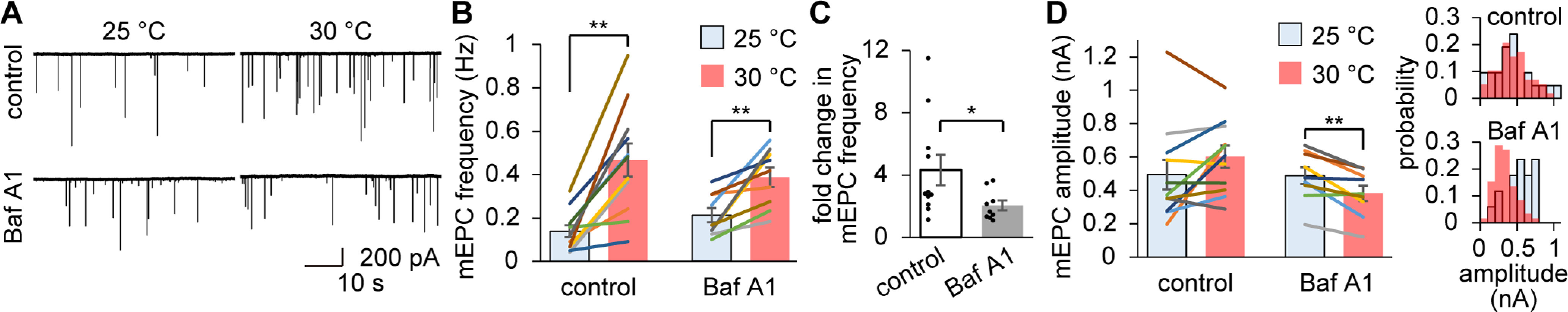
The temperature-dependent increase in mEPC events was greatly impaired by the inhibition of transmitter refilling. ***A***, Traces of mEPC recorded at 25°C or 30°C from Baf A1-treated or Baf A1-untreated (control) muscles. ***B***, Temperature dependency of mEPC frequency in control (*n* = 11 cells from 11 fish) and Baf A1-treated (*n* = 9 cells from 9 fish) conditions. In both conditions, temperature rise resulted in a significant increase in the mEPC frequency (**p < 0.01, paired *t* test). ***C***, Fold change in mEPC frequency resulting from temperature rise. Baf A1 treatment significantly decreased the temperature dependency of mEPC frequency (*p < 0.05, unpaired *t* test). ***D***, Temperature dependency of mEPC amplitude analyzed from the same datasets as in ***B***. Temperature rise significantly reduced mEPC amplitude only in the Baf A1 condition (**p <0.01, paired *t* test). Right panels, Histograms of mEPC amplitudes recorded from representative cells. Error bars indicate ±SEM.

To test whether the early-onset component of the observed spontaneous SV labeling matches the spontaneous release recorded as mEPCs in electrophysiology, we examined the temperature dependency of mEPCs. When temperature was raised from 25°C to 30°C within 20 min of TTX application, mEPC frequency increased dramatically (Fig. 5*A*,*B*, control) with an average fold change of 4.3 ± 1.0 (Fig. 5*C*, control). This temperature dependency was substantially larger than that of SV labeling (Fig. 4*D*). This discrepancy raised two possibilities. First, HaloTag labeling may fail to detect a subpopulation of spontaneous fusion activated at 30°C. Second, mEPCs recorded at 30°C may represent a higher reuse rate of SVs that have already undergone initial HaloTag labeling. To test these two possibilities, the temperature sensitivity of mEPCs was assessed immediately after Baf A1 treatment (4 μm for 2.5 min), which blocks SV re-acidification (Fig. 2*E*) and thus prevents SV refilling with ACh. Average fold change of mEPC frequency under Baf A1 was significantly reduced compared with that of control (2.1 ± 0.3 for Baf A1; Fig. 5*A–C*), which supports that the temperature-dependent increase in mEPC frequency relied more on the SV reuse requiring the ACh refilling. Moreover, mEPC amplitude was significantly reduced with increasing temperature after Baf A1 treatment (Fig. 5*D*). Since permanently charged ACh does not leak from the SVs (Edwards, 2007), the decreased mEPC amplitude implies that insufficiently refilled SVs are exocytosed. This finding again supports the higher reuse rate at 30°C. These measurements demonstrated the significant contribution of repeated SV reuse to the miniature recording, which accounts for the discrepancy between the two methods and thus supports the validity of HaloTag-based spontaneous SV labeling.

### TeNT inhibited the late-onset population of spontaneous SV fusion

**Figure 6.**
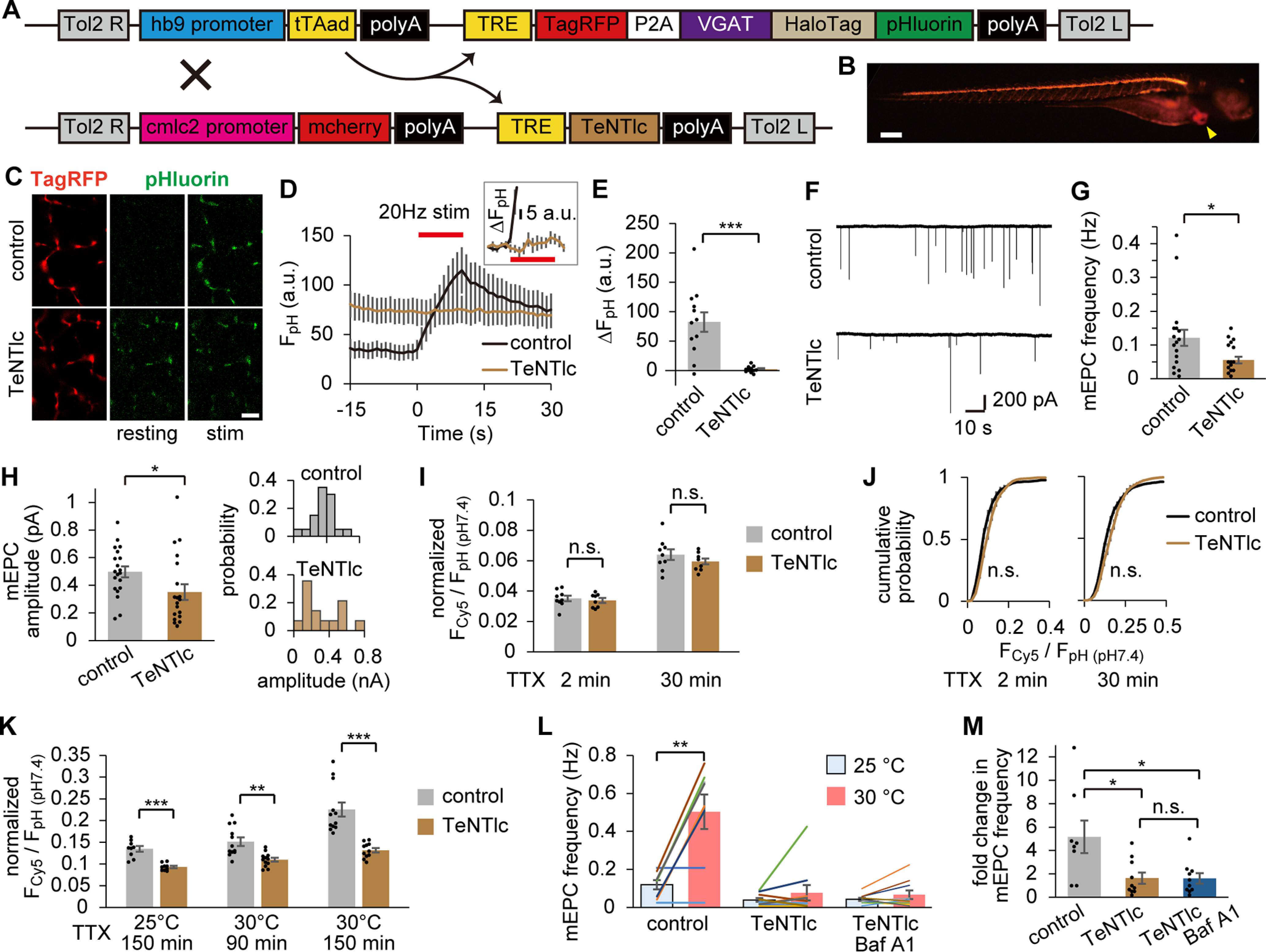
TeNT impaired the late-onset component of spontaneous SV labeling. ***A***, Diagram represents transgene constructs for Tg (*hb9:tTAad, TRE:TagRFP-P2A-VpHalo*) and Tg (*cmlc2:mcherry, TRE:TeNTlc*) zebrafish. In the DTg fish, TeNTlc was expressed in addition to TagRFP and VpHalo in motoneurons via the Tet-Off system driven by the hb9 promoter. mcherry expressed in the heart by the cmlc2 promoter was used as a marker of the transgene. ***B***, Fluorescent stereoscopic image of TagRFP and mcherry (arrowhead) in the DTg fish at 4 dpf. Scale bar, 200 μm. ***C***, Confocal live images of pHluorin before (resting) and after (stim) APs at 20 Hz for 10 s in control or DTg fish. TagRFP fluorescence in the same region is also shown. Scale bar, 10 μm. ***D***, pHluorin fluorescence in response to APs at the NMJs in control (*n* = 12 experiments from 6 fish) or DTg fish (*n* = 11 experiments from 6 fish). Inset, A magnified view of the fluorescence increase of DTg. ***E***, Increases in fluorescence at the end of APs were significantly decreased in DTg fish (***p < 0.001, unpaired *t* test). ***F***, Traces of mEPCs recorded from control or DTg fish. ***G***, ***H***, mEPC frequency (***G***) and amplitude (***H***) in control (*n* = 20 cells from 10 fish) or DTg fish (*n* = 20 cells from 10 fish). DTg fish showed significant reduction in both frequency (*p < 0.05, unpaired *t* test) and amplitude (*p < 0.05, unpaired *t* test). Right panels, Histograms of mEPC amplitudes recorded from representative cells. ***I***, Spontaneously labeled fraction in control or DTg fish at 2 min (*n* = 9 or 8 fish, respectively) or 30 min (*n* = 9 or 9 fish, respectively) at 25°C. No significant difference was seen in 2 and 30 min labeling (*p* = 0.61 and 0.28, respectively, unpaired *t* test). ***J***, Cumulative probability histogram of F_cy5_/F_pH (pH7.4)_ at individual ROIs from the data analyzed in ***H***. No significant difference was seen both in 2 and 30 min labeling (*p* = 0.06 and 0.12, respectively, Kolmogorov–Smirnov test). ***K***, Spontaneously labeled fraction in control or DTg fish over 150 min at 25°C (*n* = 9 or 9 fish, respectively), 90 min at 30°C (*n* = 12 or 12 fish, respectively), or 150 min at 30°C (*n* = 12 or 11 fish, respectively). The labeled fraction in DTg fish was significantly smaller than that of control in all conditions (**p < 0.01, ***p < 0.001, unpaired *t* test). ***L***, Temperature dependency of mEPC frequency in control (*n* = 8 cells from 8 fish), DTg (*n* = 10 cells from 10 fish), and Baf A1-treated DTg (*n* = 10 cells from 10 fish). Temperature rise (from 25°C to 30°C) significantly increased the mEPC frequency in control (**p < 0.01, paired *t* test). ***M***, Fold change in mEPC frequency resulting from temperature rise. In DTg, the temperature dependency of mEPC frequency was significantly decreased compared with control (*adjusted *p* < 0.05, one-way ANOVA followed by Bonferroni–Holm test). Baf A1 treatment had no effect in DTg (adjusted *p* = 0.98, one-way ANOVA followed by Bonferroni–Holm test). Error bars indicate ±SEM.

To gain further insight into the difference between the early- and late-onset components of spontaneous SV fusion, we examined the effects of TeNT, a clostridial toxin that cleaves VAMP1 and VAMP2. Reports in multiple systems have shown that the application of TeNT or genetic ablation of VAMP2 nearly eliminated evoked release, whereas a substantial fraction of miniature currents remained unaffected (Mellanby and Thompson, 1972; Sweeney et al., 1995; Schoch et al., 2001). We generated a Tg zebrafish carrying a transgene containing the TeNTlc under the control of a Tet-inducible promoter (TRE). When this Tg was crossed with VpHalo-expressing Tg, the obtained double Tg (DTg) expressed TeNTlc specifically in motoneurons (Fig. 6*A*,*B*). To examine the effect of TeNT on evoked release at the NMJs, the pHluorin response to APs (20 Hz, 10 s) was imaged. The baseline fluorescence in DTg was higher compared with the control, suggesting that either the surface probes or the SV luminal pH was increased in DTg (Fig. 6*C*,*D*). Regardless of the increased baseline, the response to APs nearly disappeared in DTg, with a 97.5% reduction in ΔF_pH_ at the end of the APs compared with the control (Fig. 6*E*). This decrement indicated that evoked release depended largely on the TeNT-sensitive v-SNAREs. In contrast, mEPCs remained relatively robust in DTg (Fig. 6*F*,*G*), with the frequency reduced to 41% compared with the control when averaged across all experiments (see also Fig. 6*L*). The amplitude of the remaining mEPCs recorded in DTg was also significantly smaller (Fig. 6*H*), which is consistent with a previous report (Bao et al., 2018). Despite these changes, a substantial population of spontaneous release in zebrafish NMJs was mediated by TeNT-resistant v-SNAREs.

Next, we tested the effects of TeNT on the HL-Cy5 labeling of spontaneous SVs. Fish were labeled with HL-Cy5 in the presence of TTX for 2 and 30 min at 25°C, which would label early-onset populations. Neither the average normalized F_Cy5_/F_pH (pH7.4)_ nor the cumulative histogram of F_Cy5_/F_pH (pH7.4)_ measured at individual ROIs was different between the two groups (Fig. 6*I*,*J*), indicating that neither the surface probes nor the early-onset population of spontaneous fusion were affected by TeNT. In contrast, when labeling was performed for 150 min at 25°C or >90 min at 30°C to evaluate the late-onset population, the labeled fraction in DTg was significantly suppressed in all conditions (Fig. 6*K*). Therefore, only the late-onset population was sensitive to TeNT. These results indicated that prolonged AP inhibition activates a molecularly distinct process for spontaneous fusion and supports the idea that the bona fide SV pool undergoing spontaneous recycling arises from the limited population observed in the early phase of AP blockade.

The results that mEPC frequency decreased in DTg whereas spontaneous SV labeling in the early phase of AP blockade remained unchanged may seem paradoxical (Fig. 6*G*,*I*). Since the number of ROIs identified in a single image of the labeling experiments were not different between the control (122 ± 3.9) and DTg groups (132 ± 4.3, *p* = 0.1, unpaired *t* test), changes in presynaptic density were unlikely. We hypothesized that the TeNT-resistant mEPCs observed in DTg were mainly mediated by virgin exocytosis, not by the repeated reuse of SVs which escapes detection by HaloTag labeling. Since temperature-dependent increase in mEPC frequency largely relied on SV reuse (Fig. 5), we examined mEPCs in DTg at the normal and increased temperature: 25°C and 30°C (Fig. 6*L*). The increase in temperature caused only a 1.7 ± 0.5-fold increase in the mEPC frequency in DTg, which was significantly smaller than in the control group (5.2 ± 1.4, Fig. 6*M*). Moreover, Baf A1 treatment of DTg did not show an additive effect on the temperature dependency (1.6 ± 0.4, Fig. 6*M*). These data supported our hypothesis.

### Spontaneous release of SVs did not arise from the resting pool

A previous study using SV labeling based on biotinylated VAMP2 in hippocampal culture reported that spontaneous fusion arose from the SV pool that did not respond to strong electrical stimulation (900 APs at 20 Hz) (Fredj and Burrone, 2009). In zebrafish NMJs, 15% of SVs were not mobilized by similar strong stimulation (1200 APs at 20 Hz), which corresponds to the resting pool by definition (Fig. 2*F*). We examined whether spontaneous fusion, which constitutes 8% of SVs (Fig. 4*D*), is included in the resting pool.

**Figure 7.**
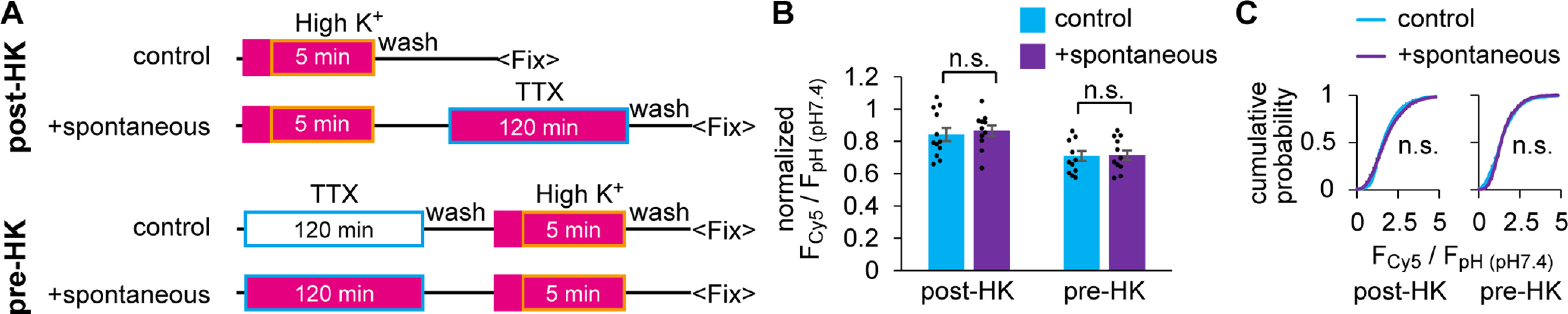
Spontaneously recycling SVs were included in the total recycling pool. ***A***, Diagram represents the timeline of the sequential labeling experiment. Spontaneous SV labeling in TTX for 120 min was performed after (post-HK) or before (pre-HK) the evoked SV labeling in 5 min HK depolarization. Fish preparations were subjected to incubation with HL-Cy5 (magenta square) followed by HK depolarization (boxed in orange) or to incubation in the presence of TTX (boxed in blue). ***B***, SV fraction labeled with HK only (control) or sequentially with both HK and TTX (+spontaneous) in post-HK labeling (*n* = 12 or 11 fish, respectively) and pre-HK labeling (*n* = 12 or 12 fish, respectively). No significant difference was observed between the two groups both in post-HK (*p* = 0.67, unpaired *t* test) and pre-HK (*p* = 0.90, unpaired *t* test) labeling. ***C***, Average cumulative probability of F_Cy5_/F_pH (pH7.4)_ at individual ROIs from the data analyzed in ***B***. No significant difference was observed between the two groups in both post-HK (*p* = 0.44, Kolmogorov–Smirnov test) and pre-HK (*p* = 0.32, Kolmogorov–Smirnov test).

Spontaneous labeling with HL-Cy5 for 120 min was performed before or after the evoked labeling by 5 min HK depolarization (Fig. 7*A*). In both conditions, spontaneous labeling did not increase the labeled fraction compared with that in the control, where SVs were labeled only during HK depolarization (Figs. 7*B* and 3*F*). Cumulative histograms of F_Cy5_/F_pH (pH7.4)_ measured at individual ROIs did not show any change (Fig. 7*C*), indicating that spontaneous release originates not from the resting pool but from a population of SVs that undergo evoked release in zebrafish NMJs.

### Spontaneously recycling SVs matched RRP

**Figure 8.**
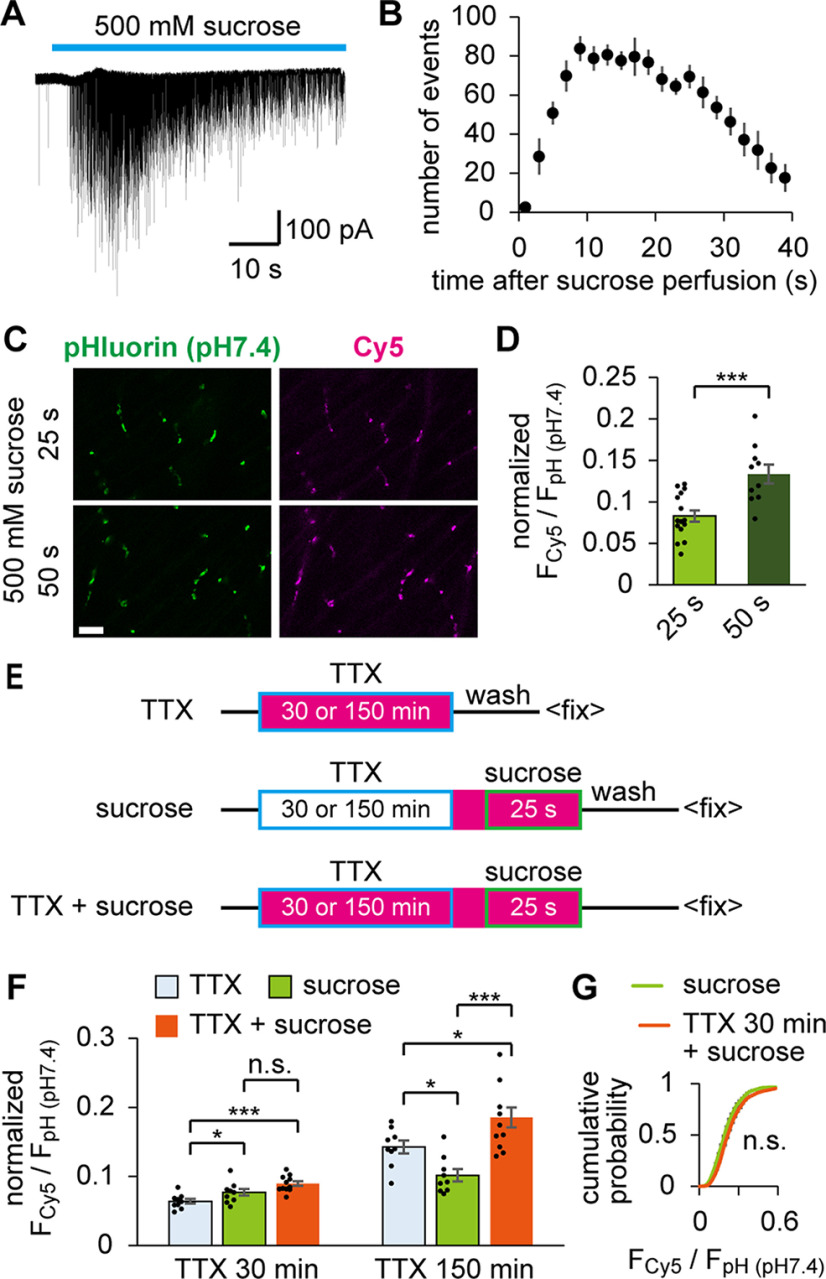
Spontaneously recycled SVs overlapped with RRP mobilized by hypertonic stimulation. ***A***, A trace of the mEPC burst recorded during perfusion of 500 mm sucrose. Unlike in cultured neurons, individual mEPCs could be resolved. ***B***, The numbers of mEPC events caused by 500 mm sucrose were counted in 2 s bins. The number of events decreased after 25 s. ***C***, Confocal images of pHluorin (pH 7.4) and Cy5 at the NMJs, where HaloTag labeling was achieved by the hypertonic stimulation for 25 or 50 s. Scale bar, 10 μm. ***D***, Labeled fractions during 25 s (*n* = 15 fish) or 50 s (*n* = 10 fish) hypertonic stimulation. Longer stimulation mobilized a larger fraction (***p < 0.001, unpaired *t* test). ***E***, Diagram represents the timeline of sequential labeling experiments. In the first group (TTX), fish preparations were incubated with HL-Cy5 (magenta) in the presence of TTX (boxed in blue) for 30 or 150 min. In the second group (sucrose), TTX treatment for 30 or 150 min was followed by sucrose stimulation for 25 s (boxed in green) in HL-Cy5. In the third group (TTX+sucrose), the TTX treatment and sucrose stimulation were both performed in HL-Cy5. ***F***, Labeled fractions obtained by the experiments shown in ***E***. When the TTX treatment was 30 min, a measurable fraction was labeled in the TTX group (*n* = 9 fish). However, the TTX+sucrose group (*n* = 11 fish) did not significantly increase the labeled fraction compared with that in the sucrose group (*n* = 10 fish, adjusted *p* = 0.08, one-way ANOVA followed by Bonferroni–Holm test). In contrast, when the TTX treatment was extended to 150 min, the TTX+sucrose group (*n* = 11 fish) significantly increased the labeled fraction compared with that in the sucrose (*n* = 9 fish) and TTX groups (*n* = 9 fish). *Adjusted *p* < 0.05, ***adjusted *p* < 0.001, one-way ANOVA followed by Bonferroni–Holm test. ***G***, Cumulative probability histogram of F_cy5_/F_pH (pH7.4)_ at individual ROIs in the TTX+sucrose or sucrose group (TTX treatment for 30 min in both groups), which were analyzed in ***F***. No significant difference was seen (*p* = 0.20, Kolmogorov–Smirnov test). Error bars indicate ±SEM.

Because spontaneously recycling SVs did not arise from the resting pool (Fig. 7), we next examined whether they overlapped with the RRP, an immediately responding component in the total recycling pool. To estimate the fraction of RRP in zebrafish NMJs, hypertonic stimulation that depletes RRP (Rosenmund and Stevens, 1996) was used. Perfusion with 500 mm sucrose caused a burst of mEPCs (Fig. 8*A*). The number of events increased during the first 10 s and reached a plateau. Events started to decrease after 25 s (Fig. 8*B*), which may represent mobilization of the recycling pool or reuse of RRP. Thus, at least 25 s of hypertonic stimulation is required to mobilize the preponderance of RRP. A similar estimate was also obtained for frog NMJs (Rizzoli and Betz, 2004).

We performed HaloTag-based SV labeling with hypertonic stimulation for 25 or 50 s and obtained 8.3 ± 0.7% and 13.4 ± 1.1% labeling, respectively, as shown by normalized F_Cy5_/F_pH (pH7.4)_ (Fig. 8*C*,*D*). These values did not contradict the estimation of early-onset spontaneously labeled SVs (Fig. 4*D*) if the latter overlapped with the RRP. Sequential labeling was performed to test this possibility. Labeling with HL-Cy5 during the 25 s sucrose stimulation was performed after TTX treatment in the presence or absence of the labeling (Fig. 8*E*). TTX treatment was set to either 30 or 150 min to clarify the effect of the late-onset component. In the 30 min TTX treatment, sequential labeling (TTX+sucrose) did not significantly increase the signal compared with that of sucrose stimulation alone (sucrose; Fig. 8*F*). Cumulative histograms of F_Cy5_/F_pH (pH7.4)_ measured at individual ROIs were not significantly different between the two groups (Fig. 8*G*), indicating that SVs dedicated to spontaneous fusion overlapped with the RRP. In contrast, when TTX treatment was extended to 150 min, sequential labeling (TTX+sucrose) resulted in an increase in the labeled fraction compared with either the spontaneous labeling alone (TTX) or the hypertonic labeling alone (sucrose; Fig. 8*F*). These results suggest that spontaneously labeled SVs maintained their RRP state, at least in the early stage of the AP blockade, whereas intermixing of SVs between the RRP and other pools was facilitated at the later stage of AP blockade.

**Figure 9.**
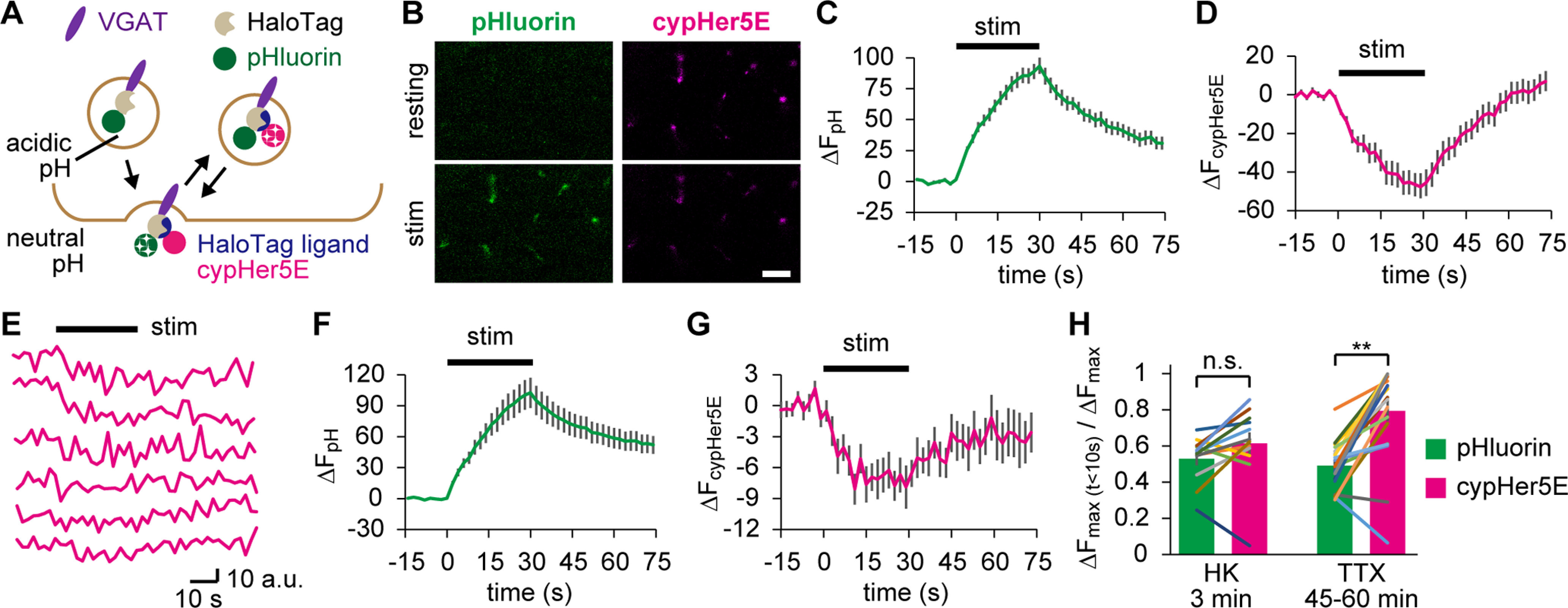
Spontaneously recycled SVs behaved like RRP vesicles in subsequent APs. ***A***, Diagram represents HaloTag labeling with HL-cypHer5E, which allows SV imaging after the initial exocytosis. ***B***, Confocal live images of pHluorin and cypHer5E at the NMJs before (resting) and after (stim) APs (20 Hz for 30 s). HL-cypHer5E was loaded by 3 min HK depolarization preceding the electrical stimulation. Scale bar, 10 μm. ***C***, ***D***, pHluorin fluorescence (***C***) and cypHer5E fluorescence (***D***) in response to APs (20 Hz for 30 s) measured at the NMJs prelabeled with HL-cypHer5E through 3 min HK depolarization (*n* = 12 experiments from 7 fish). The cypHer5E fluorescence, which is maximum at acidic pH, is not photostable; thus, its photobleaching was corrected (see Materials and Methods). ***E***, cypHer5E fluorescence in response to APs (20 Hz for 30 s), where HL-cypHer5E was preloaded during 45-60 min incubation in TTX. Each trace is from an individual experiment. ***F***, ***G***, pHluorin fluorescence (***F***) and cypHer5E fluorescence (***G***) in response to APs (20 Hz for 30 s) measured at the NMJs preloaded with HL-cypHer5E during 45-60 min incubation in TTX (*n* = 20 experiments from 16 fish). ***H***, The fraction of SVs exocytosed in the first 10 s of electrical stimulation was calculated for preloaded SVs (cypHer5E) and the total SVs (pHluorin). Preloading was performed either by HK stimulation or incubation in TTX. SVs preloaded in TTX fused significantly faster than that in total SVs (TTX 45-60 min; **p < 0.01, paired *t* test), which was not the case in SVs preloaded by HK (HK 3 min; *p* = 0.08, paired *t* test). Error bars indicate ±SEM.

SVs in RRPs are expected to be exocytosed in response to APs with a faster time course. To examine whether the spontaneous recycling pool displayed such characteristics, we replaced the dye of the HaloTag ligand with cypHer5E, which has a fluorescence spectrum similar to that of Cy5 but exhibits pH sensitivity with a pK_a_ similar to that of pHluorin. Although cypher5E and pHluorin respond to opposite changes in pH, both fluorophores report activity-evoked SV recycling in a similar fashion when targeted to the SV lumen (Hua et al., 2011a). In Tg zebrafish expressing VpHalo, SVs tagged with HL-cypHer5E on initial exocytosis showed a fluorescence drop on subsequent rounds of exocytosis, whereas exocytosis of total SVs could be visualized by a jump in pHluorin fluorescence (Fig. 9*A*). We labeled the Tg fish with HL-cypHer5E either by 3 min HK depolarization (Fig. 9*B–D*) or by 45-60 min TTX (Fig. 9*E–G*), to label total recycling pools and the spontaneous recycling pool, respectively. We compared the dynamics of SVs on subsequent APs using both pHluorin and HL-cypHer5E.

When labeled in the HK condition, cypHer5E appeared in almost all boutons (Fig. 9*B*), and fluorescence from both pHluorin and cypHer5E continued to respond until APs (20 Hz for 30 s) ceased (Fig. 9*C*,*D*), indicating that SVs prelabeled in HK depolarization corresponded to the total recycling pool during subsequent AP firing.

In contrast, when labeled in the TTX condition, cypHer5E fluorescence was dim because of their smaller pool size. Nevertheless, APs gave clear responses frequently, which seemed to coincide with the initial phase of the stimulation (Fig. 9*E*). Averaged data of all experiments indicated that cypHer5E fluorescence reached a plateau at 10 s, while pHluorin fluorescence measured at the same boutons continued to increase throughout the 30 s APs (Fig. 9*F*,*G*). To statistically compare the rate of SV fusion between total SVs and SVs labeled in either HK or TTX, increases in fluorescence during the first 10 s (ΔF_max (t<10s)_) were divided by the maximum fluorescence during the 30 s APs (ΔF_max_) for both pHluorin and cypHer5E. The values in the HK prelabeling group were not significantly different between the two reporters, whereas in the spontaneous prelabeling group, cypHer5E showed a significantly larger value (Fig. 9*H*). These results indicated that spontaneously recycled SVs mobilized faster than other SVs on APs, which strongly supports that spontaneously recycled SVs constitute RRP vesicles.

## Discussion

**Figure 10.**
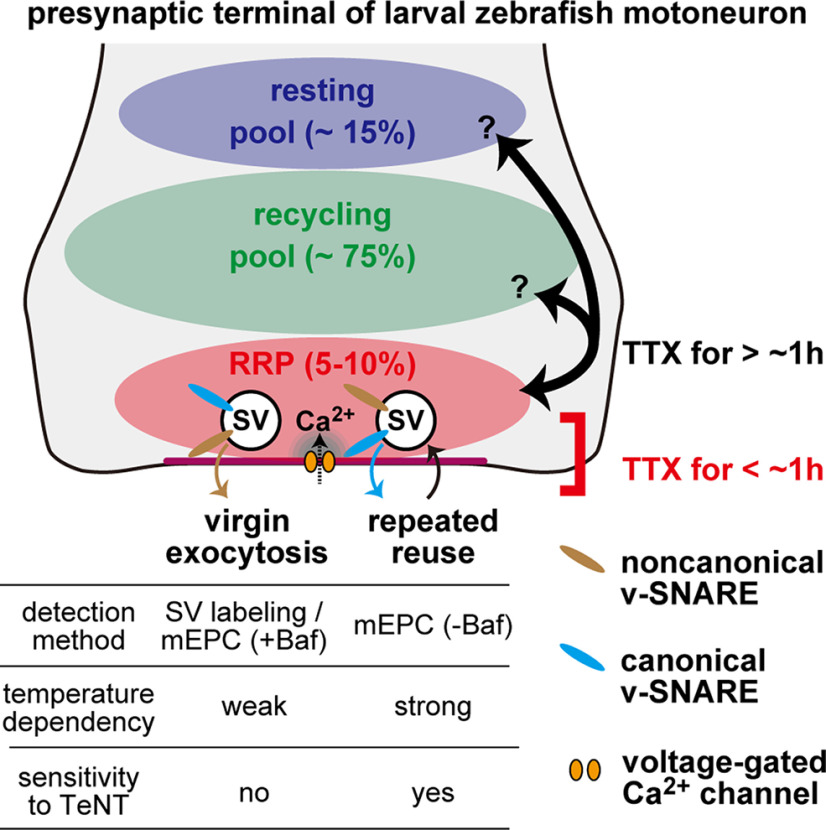
Summary diagram of spontaneous SV fusion at larval zebrafish neuromuscular synapses. Three pools of SVs are depicted: resting pool, recycling pool, and RRP. In the early phase of TTX treatment (<1 h), spontaneous SV fusion is mobilized from the RRP with two distinct modes: virgin exocytosis with a slow time course (τ = 45 min at 25°C) and repeated reuse of the same SVs at a higher rate. The differences between the two modes of fusion are highlighted in the table at bottom. SVs equipped with both canonical and noncanonical v-SNAREs are involved in the two modes, although their dependency on the noncanonical v-SNARE is not identical. They may also be different in their coupling to the voltage-gated Ca^2+^ channels. SVs in RRP are intermixed with those in other pools after a prolonged (>1 h) TTX treatment.

Spontaneous release is presumed to result from the fluctuation of local [Ca^2+^] at presynaptic terminals, in which some SVs are docked to the active zone membrane, ready for exocytosis on the transient rise of Ca^2+^ following APs. It was therefore a natural assumption that the SVs used for spontaneous release originated from RRP. This notion was supported by electrophysiological studies regarding the role of Ca^2+^ on evoked and spontaneous release, with a caveat that they potentially rely on distinct Ca^2+^ sensors (Schneggenburger and Rosenmund, 2015). However, direct evaluation of SV recycling using genetically encoded optical probes, particularly applied to cultured neurons, contradicted this hypothesis (Sara et al., 2005; Groemer and Klingauf, 2007; Fredj and Burrone, 2009; Chung et al., 2010; Hua et al., 2010; Wilhelm et al., 2010). In this study, we provided the first imaging-based evidence supporting the same origin of spontaneous release and RRP (Fig. 10) by applying two genetic tools in intact NMJs of larval zebrafish, which has been intensively used for *in vivo* analysis of synaptic transmission (Brehm and Wen, 2019).

We introduced pHluorin and HaloTag into the SV lumen, which enabled us to separate distinct SV pools with a quantification of each pool size (Figs. 1–4). In particular, the HaloTag ligand with a pH-sensitive indicator, newly used to visualize spontaneously recycled SVs, was beneficial because we could isolate their dynamics during subsequent evoked activity (Fig. 9). The use of VGAT as a carrier of the optical probes was also advantageous because of its extremely low surface fraction (Fig. 4*D*). Other carrier SV proteins, such as VAMP2 and Syt1, whether endogenous or overexpressed, have a relatively large surface pool, which also participate in SV recycling (Hua et al., 2011a) and may complicate the interpretation of the origin of spontaneous release.

TTX treatment, a manipulation required to isolate spontaneous fusion, changed the characteristics of spontaneous SV recycling within 1-2 h with regard to their temperature sensitivity and TeNT resistance (Figs. 4, 6, and 10). This late-onset process is reminiscent of homeostatic scaling of presynaptic functions that compensate for the perturbation of neural signaling (Davis and Muller, 2015), in which the remodeling of SV pools is involved (Kim and Ryan, 2010; Muller et al., 2012). Indeed, intermixing of SVs between RRP and other pools was observed in this process (Figs. 8*F* and 10). Although further investigations are required to prove whether it is really a homeostatic plasticity, this finding led us to conclude that spontaneous fusion under physiological conditions, that is, in the absence of TTX, always originates from the population that saturated at 8.2% of the total SV (Figs. 4*D* and 10).

We believe that the use of intact NMJs is critical for our observation. Recent studies have shown that axonal transection, usually unavoidable in adult NMJ preparations, initiates molecular signaling that actively causes degeneration of distal axons (Conforti et al., 2014). Because such signaling may obscure other cellular processes, intact synapses are an ideal system for studying physiological phenomena in which plasticity may be involved. Moreover, in zebrafish NMJs at 4-5 dpf, constitutively trafficking organelles required for synaptogenesis was negligible (Fig. 4*D*), indicating that the NMJs at this stage were already functionally mature, as shown by previous studies (Saint-Amant and Drapeau, 1998; Nguyen et al., 1999). Since constitutive fusion occurs independent of AP firing and obscures bona fide spontaneous fusion (Truckenbrodt and Rizzoli, 2014), its absence is important. The situation in cultured neurons may be different, where the maturity of synapses likely varies among cultures because synaptogenesis continues over several weeks with a rate depending on the neuronal density (Biffi et al., 2013).

SVs labeled during the early phase of AP blockade were resistant to TeNT (Fig. 6*I*,*J*), which cleaves neuronal v-SNAREs, VAMP1 and VAMP2, both of which are required for evoked release at the NMJs (Liu et al., 2019). Several studies have shown that spontaneous release is driven by noncanonical v-SNAREs, such as VAMP4 (Lin et al., 2020), VAMP7 (Hua et al., 2011b; Bal et al., 2013), and Vti1a (Ramirez et al., 2012; Crawford et al., 2017). Since these v-SNARE genes are all conserved in the zebrafish genome, it is possible that one or several of these noncanonical v-SNAREs drive the spontaneous release in NMJs (Fig. 10). Nevertheless, spontaneously recycled SVs were also mobilized during the subsequent AP-dependent exocytosis (Fig. 9*E–H*), which relied mostly on TeNT-sensitive v-SNAREs (Fig. 6*E*). Therefore, our findings suggest that SVs may be equipped with proteins that support both evoked and spontaneous release (Fig. 10). This idea contradicts several studies that have shown that noncanonical v-SNAREs fused with pHluorin are preferentially used for spontaneous fusion and are reluctant to respond to evoked activity (Hua et al., 2011b; Ramirez et al., 2012; Bal et al., 2013; Lin et al., 2020), leading to the development of a heterogeneous SV pool model with distinct protein compositions. However, recent analyses of SV recycling using novel lipid-based tracers have argued against this idea (Kahms and Klingauf, 2018), drawing attention to the effect of overexpression of noncanonical v-SNAREs, whose copy number is inherently low, for example, two copies of Vti1a per average SV from adult rat brains (Takamori et al., 2006). The paucity of noncanonical v-SNAREs raises the question of whether all SVs can accommodate the TeNT-resistant form of spontaneous release. Considering that only SVs that matched to RRP were used for spontaneous release in our preparation, it is possible that SVs with a complete set of proteins are preferentially recruited to the RRP, although the sorting mechanism remains unidentified.

The frequency of mEPCs at a single NMJ of larval zebrafish was estimated to be ∼0.01 Hz at 25°C since mEPCs were recorded at 0.14 Hz (Fig. 5*B*) from a muscle receiving 12-15 synaptic inputs (Wen et al., 2016b; Brehm and Wen, 2019), which is in line with reports in *Drosophila* NMJs or cultured neurons (Murthy and Stevens, 1999; Melom et al., 2013). In addition to the slow mobilization of virgin SVs (τ = ∼45 min) revealed by HaloTag labeling (Fig. 4*D*), repeated reuse of the same SVs that eludes detection with labeling also occurs with a relatively fast time course and contributes to electrophysiological recording of spontaneous transmission (Ertunc et al., 2007). The reuse of SVs is strongly dependent on the temperature (Fig. 5). The natural habitat of zebrafish close to 30°C therefore suggests that SV reuse is more important in determining the frequency of mEPCs (Fig. 10). Moreover, mEPC frequency in TeNTlc DTg was significantly reduced even at 25°C (Fig. 6*G*,*L*), while the spontaneous labeling did not change after 30 min TTX treatment (Fig. 6*I*). Although we cannot rule out the possibility that HaloTag-based SV labeling is less sensitive than electrophysiological techniques, we are inclined to the interpretation that a substantial fraction of mEPCs detected in baseline condition is mediated by repeated reuse of SVs, dependent on the TeNT-sensitive, canonical v-SNAREs (Figs. 6*M* and 10). It is worth noting here that the two observed modes of spontaneous release (i.e., virgin release and reuse) arise from a single pool, with characteristics similar to the RRP.

In the current model (Fig. 10), it remained undetermined whether noncanonical v-SNAREs present in RRP SVs function in response to the APs. We propose they do respond to some extent because a small pHluorin response to high-frequency AP firing was observed in TeNTlc DTg, although its proportion to the total SVs could not be estimated because of the elevated baseline (Fig. 6*D*). Notably, this response occurred with a discernible delay after the stimulus, suggesting that noncanonical v-SNAREs are loosely coupled to presynaptic Ca^2+^ transients during AP firings. Therefore, noncanonical v-SNAREs do not make SVs “readily releasable.” Interestingly, spontaneous SV release can be separated in two distinct categories based on the Ca^2+^ dependency (Schneggenburger and Rosenmund, 2015), and Ca^2+^ sensors interacting with noncanonical v-SNAREs may be different from those mediating the fast transmission (Raingo et al., 2012). In this context, involvement of Ca^2+^ on the two modes of spontaneous release awaits clarification by further research (Fig. 10).
